# The use of mobile phones in polio eradication

**DOI:** 10.2471/BLT.15.163683

**Published:** 2015-11-27

**Authors:** Abdul Momin Kazi, Lubna Ashraf Jafri

**Affiliations:** a Department of Paediatrics and Child Health, Aga Khan University, Stadium Road, PO Box 3500, Karachi, 74800, Pakistan.

The Global Polio Eradication Initiative, established in 1988, has led to the immunization of 2.5 billion children against the disease. In 2014, only 359 cases of poliomyelitis (polio) were reported worldwide, which is a fraction of the estimated 350 000 children in 125 countries who were paralyzed annually by the poliovirus before 1988. There are only two remaining countries where polio is still endemic – Afghanistan and Pakistan, while circulating vaccine-derived poliovirus is still causing outbreaks in others, such as Guinea, Madagascar and Ukraine. Spread of the poliovirus from the two remaining endemic countries to Iraq, Israel, the Syrian Arab Republic and other vulnerable countries is a continuing threat. Until polio transmission in endemic countries is interrupted, the whole world remains at risk.

Despite recent developments and improvements in polio immunization campaigns in high-risk countries, major challenges remain. The foremost factor responsible for low immunization coverage is poor security, particularly in Afghanistan and Pakistan. Other contributing factors include a lack of population-based immunization registries and electronic records, overestimating vaccination coverage, human error, drop-outs from immunization campaigns, low demand for vaccination in the population and a lack of awareness of, or education about, immunization among parents.

Mobile health (mHealth) is defined as medical and public health practices supported by mobile electronic devices. Recently there has been a dramatic increase in mobile phone use and there are now around 7 billion mobile phone subscribers globally, 89% of whom live in developing countries. Thanks to the portability of mobile phones, more people can be reached by phone than through the Internet and they can be contacted quickly and inexpensively wherever they are located. Even a substantial proportion of those living on less than 1 United States dollar a day have access to mobile phones and short message service (SMS) text messages. Mobile phones are thus changing the mode of communication among people globally. Importantly, mobile phone use has also increased in countries with a high risk of polio: good examples are Nigeria and Pakistan, where there were around 170 and 140 million mobile phone subscribers, respectively, in 2014.^1^^,^^2^

Given increased mobile phone use, mHealth interventions now have great potential to support the polio eradication programme. In fact, they have been used in the recent past. In Pakistan, for example, an SMS-based service was provided in 2010 that enabled parents to send a free text message to report areas missed by the national polio control programme, with the result that a polio immunization team was dispatched shortly after.^3^ In addition, text messages about the polio campaign were sent to more than 8 million mobile phone subscribers to raise awareness, especially in high-risk areas. In another study in Pakistan, smart phones incorporating a geographical information system were used to conduct a survey of mobile phone access and use among caregivers of children younger than five years in randomly selected representative clusters in high-risk towns (i.e. administrative areas) within Karachi where polio cases had previously been detected. The data obtained were linked to an automated text message system used to monitor supplementary immunization activities in Karachi.^4^

In Nigeria, two pilot studies in 2011 demonstrated that creating maps using a geographical information system and integrating them into the detailed planning of polio vaccination campaigns provided feedback in real time: they gave information about the performance of the vaccination teams and helped identify missed or partially covered settlements.^5^ One component of another study in Nigeria included sending SMS text reminders to improve surveillance of acute flaccid paralysis (Oluwasegun Adegoke, personal communication, 2015). In the Syrian Arab Republic, an application for mobile phones operating on the global system for mobile communications (GSM) standard was developed in 2014 to track the number of polio vaccination doses children had received and to create electronic records of immunization in a central database – the application was used by community health workers administering the polio vaccine.^6^

In Somalia, a mobile phone-based health promotion project was implemented in 2013 to support polio prevention and control. The project involved two complementary components: (i) pre-emptive community education on polio prevention using interactive SMS text messages; and (ii) the distribution of soap, water containers and household water treatment items important for polio prevention, all obtained by redeeming vouchers provided in SMS messages. One benefit of receiving information via mobile phones is that it can be tailored to the specific population and viewed whenever convenient, in contrast to less flexible radio and television broadcasts.^7^ In India, the dial tone on mobile phones was replaced during anti-polio campaigns with a recorded message that reminded the public of National Immunization Day whenever a call was dialled. This strategy worked very well and the campaign was successful.^8^ Similarly, a community-level mHealth project in India provided community health workers with a mobile phone toolkit that helped them: (i) access and organize immunization data on all children; (ii) track missed vaccinations; and (iii) design house-to-house immunization strategies for areas where mobile phone connectivity was limited.^9^

Although numerous studies and projects involving mHealth have been implemented to aid polio eradication, the majority were either pilot studies, activities carried out on a single occasion or projects limited to a particular geographical location. It is essential that policies are developed to scale up the most successful approaches to support polio eradication, especially in areas with a high risk of the disease or poor security. Mobile phone text messages in local languages and messages with graphical images could be used educationally and as reminders about polio vaccination and surveillance. Such messages might bring about the behavioural changes necessary in the general public and health-care providers. 

Interactive voice response technology and voice messages are other options that could be used to communicate with populations where there is a low level of literacy, in particular, and that could help spread awareness at the grassroots level. Playing a catchy jingle or ringtone during immunization campaigns could also help remind children’s caregivers about polio vaccination. One advantage that both text messages and voice-based communications have over more conventional communication methods is that they can be easily, quickly and cheaply converted into different versions to suit the local context or language. For example, a voice message from a religious leader advocating polio immunization could be sent in a conservative society, whereas a similar message from a celebrity could be disseminated in other settings. Another benefit is that two-way text messaging and interactive voice responses can be used for monitoring immunization campaigns and for immunization surveillance. The added incentive of free mobile phone airtime or a voucher could further enhance an mHealth intervention if the programme required it.

More effort should be invested in exploring the use of geographical information systems on Internet-enabled devices for planning and monitoring polio immunization campaigns. Global positioning system (GPS)-enabled mobile phones can help identify, in real time, the location at which data is captured on the phone. In addition, it may be possible to collate data gathered on mobile devices by geographical location, which could be identified from information about the position of the local mobile phone tower or from other positional information on the phone. Collecting data in this way would be more efficient and cost-effective than using conventional monitoring and evaluation techniques, such as phone surveys. These data collection systems could be connected to a local or central database or data could be stored electronically for subsequent retrieval or for use in immunization monitoring or promotion. However, consent and privacy of the population covered should be a priority, as the information shared can be misused. This can be a limitation as people might not want their location to be tracked, especially in security-compromised areas.

The role of mobile devices for promoting vaccination campaigns and for monitoring health-care providers should also be explored further. These innovative approaches are equally important for routine immunization programmes and for planning and implementing the transition from polio immunization campaigns to routine polio immunization.

In addition, mHealth has great potential for enabling health-care providers involved in polio immunization programmes and polio health-care services to establish direct connections with parents and caregivers, thereby bypassing gaps in communication and other obstacles. However, there is still no clear policy on the application of mHealth to polio eradication and it is essential that all those involved work together to develop a common strategy. Interventions based on mHealth could be important in areas with poor security, war zones, hard-to-reach locations and places where there are myths about the dangers of polio programmes and little trust in immunization. In some locations, though, it can be difficult to acquire the mobile phone numbers of the relevant members of the population. National registries of the mobile phone numbers of caregivers of children younger than five years should be established, particularly in security-compromised areas with a high disease risk. In these areas, mass communication and immunization promotion based on these registries could play a central role in polio eradication. In addition, policies guiding the use of information acquired through mobile phone vendors or government agencies must be established: privacy and confidentiality are essential given the emerging threat of cybercrime. Innovative, cost-effective mHealth interventions are required to change behaviour and thereby improve both polio immunization and routine immunization uptake and coverage. There is a need for research into the cost–effectiveness, implementation and scalability of mHealth tools, applications and programmes that will help make the eradication of polio a reality.
